# Molecular Regulatory Mechanisms Affecting Fruit Aroma

**DOI:** 10.3390/foods13121870

**Published:** 2024-06-14

**Authors:** Haifei Lu, Hongfei Zhao, Tailin Zhong, Danwei Chen, Yaqiong Wu, Zhengwan Xie

**Affiliations:** 1 College of Urban Construction, Zhejiang Shuren University, Hangzhou 310015, China; luhaifei@zjsru.edu.cn (H.L.); hfzhao@zjsru.edu.cn (H.Z.); garden168zhong@163.com (T.Z.); vivi7415@163.com (D.C.); 2 Institute of Botany, Jiangsu Province and Chinese Academy of Sciences (Nanjing Botanical Garden Mem. Sun Yat-Sen), Jiangsu Key Laboratory for the Research and Utilization of Plant Resources, Nanjing 210014, China; 3 College of Forestry and Grassland, Nanjing Forestry University, Nanjing 210037, China; 4 School of Tea and Coffee, Puer University, Puer 665000, China

**Keywords:** aroma substance, synthesis pathway, structural gene, transcription factor, variety improvement

## Abstract

Aroma, an important quality characteristic of plant fruits, is produced by volatile organic compounds (VOCs), mainly terpenes, aldehydes, alcohols, esters, ketones, and other secondary metabolites, in plant cells. There are significant differences in the VOC profile of various fruits. The main pathways involved in the synthesis of VOCs are the terpenoid, phenylalanine, and fatty acid biosynthesis pathways, which involve several key enzyme-encoding genes, transcription factors (TFs), and epigenetic factors. This paper reviews the main synthetic pathways of the main volatile components in fruit, summarizes studies on the regulation of aroma formation by key genes and TFs, summarizes the factors affecting the fruit aroma formation, describes relevant studies on the improvement of fruit flavor quality, and finally proposes potential challenges and prospects for future research directions. This study provides a theoretical basis for the further precise control of fruit aroma quality and variety improvement.

## 1. Introduction

Appearance and internal quality are important indices for evaluating fruit quality. Moreover, the inherent aroma is an important index for evaluating the quality of fresh fruit commodities and is composed of volatile organic compounds (VOCs) [1,2]. Fruit aroma is composed of water-soluble and fat-soluble VOCs that can freely pass through the lipid layer of the olfactory cell membrane, creating signals to the brain olfactory center. Not all volatile substances contribute to the production of fruit aroma, and only when substances reach a specific threshold will they have an impact on the fruit aroma. Fruit aroma is a mixture of various volatile compounds, mainly terpenes, aldehydes, alcohols, esters, and ketones [3,4]. The aroma components of fruits are influenced by internal genetic factors and external environmental factors [2,4]. The intensity of the aroma is determined by the composition, content, and quantity of key substances. The aroma components of fruits include unique volatile compounds, and there are great differences in aroma components among different species, within the same species, and among different varieties [5,6]. For example, more than 979 VOCs have been identified in strawberries [7], over 200 aroma compounds have been identified in citrus fruits [8], and approximately 100 different aroma compounds have been identified in peaches [6].

In the aroma of fruits, VOCs with specific qualitative and quantitative patterns act independently of each other and co-operate with each other to produce a broad range of aromas and confer different aroma characteristics on different types of fruits [9]. Studies have shown that tomatoes, watermelon, and lemon typically exhibit an ‘herbaceous aroma’, a unique odor formed by volatile compounds derived from alcohols, aldehydes, and carotenoids [10,11]. Bananas, on the other hand, exhibit an ‘ester odor’, which is attributed to different volatile esters [12]. In addition, Forney et al. [13] used solid-phase microextraction (SPME) to separate and analyze the aroma volatiles of wild and cultivated blueberry fruits. Esters, aldehydes, and terpenes were detected by GC–O; wild blueberry fruits contain a relatively high amount of esters, while cultivated blueberries contain a high amount of aldehydes. Hence, the aroma components of fruits vary among the different varieties.

With an abundance of various fruits, the improvement of consumer quality of life, and increasing demand, there has been a pursuit of quality changes from appearance and taste to inner and recessive qualities, and consumers are paying increasing attention to the aroma quality and nutritional value of fruits. Aroma is one of the most important factors affecting the quality of fruits and their processed products. This factor is influenced by the fruit genotype, growth environment, and their interaction. In recent years, exploring the regulatory mechanism of aroma formation has become a popular research field in the fruit industry [2,3,4,5]. Previous studies have found some major compounds, potential mechanisms, and biosynthetic pathways of lipids and terpenoids that influence the volatile components of fruits [2,5], including the peach [4], pear [6], and strawberry [7]. This review rough starts with the keywords such as aroma substation, synthesis pathway, and fruits, conducting a rough search and selectively focusing on the most cited or latest research fields in recent years, summarizing the main synthesis, regulatory pathways, and molecular mechanisms of fruit aroma compounds. In addition, the potential problems are identified, and future research directions are proposed in this study to serve as a reference for cultivating fruits with good quality and strong aromas.

## 2. Main Volatile Components

**Figure 1:**
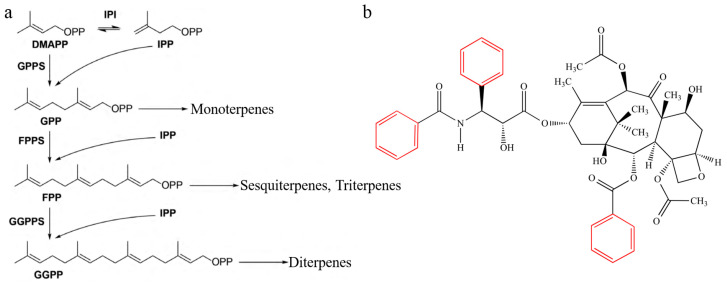
Main chemical structures of terpenoids (**a**) and benzene compounds (**b**). The red color represents the chemical structure skeleton.

Plant aroma VOCs are diverse in nature and can be classified into terpenes (Figure 1a), benzene rings (Figure 1b), and fatty acid derivatives based on their structure [9]. Terpenes are the largest class of VOCs and consist of over 550 compounds [14] derived from two common C5 precursor compounds, isopentenyl diphosphate (IPP) and its allyl isomer dimethylallyl diphosphate (DMAPP) [15]. Phenylpropanoids, containing the aromatic amino acid L-phenylalanine (Phe), are the second most common VOCs in plants. Phe is produced via two branching pathways, the shikimic acid pathway and the Phe pathway, and is linked to the central carbon metabolism. Fatty acid derivatives, including methyl jasmonate and (Z)-3-hexenol, are the third class of plant VOCs. These VOCs are all derived from C18 unsaturated fats, linolenic acid, or linoleic acid. There were significant differences in the types of aroma components and the main aroma-active components among the fruits of different species (Table 1). For example, the main aroma components of ‘Fuji’ apples at maturity are n-butyl 2-methylacetate and hexyl acetate [16]; those of bananas are 2-methylpropyl butyrate and orthonitrophenyl-beta-d-fucopyranoside [17]; those of kiwifruit are ethyl butyrate, (E)-2-hexene-1-ol, and (e)-2-hexene aldehyde [18].

## 3. Main Synthetic Pathways

### 3.1. Terpenoid Synthesis Pathway

**Figure 2:**
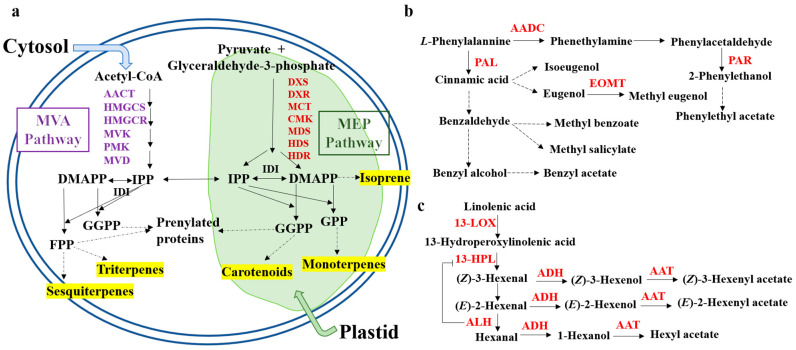
Synthetic pathways of terpenoids (**a**), phenylpropane (**b**), and fatty acid derivatives (**c**) related to the formation of aroma components. AACT, acetyl-CoA C-acetyltransferase; HMGCS, hydroxymethylglutaryl-CoA synthase; HMGCR, hydroxymethylglutaryl-CoA reductase; MVK, mevalonate kinase; PMK, phosphomevalonate kinase; MVD, mevalonate diphosphomevalonate decarboxylase; DXS, 1-deoxy-D-xylulose-5-phosphate synthase; DXR, 1-deoxy-D-xylulose-5-phosphate reductoisomerase; HDS, 4-hydroxy-3-methylbut-2-enyl-diphosphate synthase; HDR, 4-hydroxy-3-methylbut-2-en-1-yl diphosphate reductase; IDI, isopentenyl-diphosphate delta-isomerase; GPP, geranyl diphosphate synthase; GGPP, geranylgeranyl diphosphate synthase; DMAPP, dimethylallyl diphosphate; IPP, isopentenyl diphosphate; AADC, aromatic L-amino acid decarboxylase; PAL, phenylalaninammo-nialyase; EOMT, eugenol *O*-methyltransferase; PAR, phenylacetaldehyde reductase; 13-LOX, 13-lipoxygenase; 13-HPL, 13-hydroperoxidase lyase; ADH, ethanol dehydrogenase; AAT, alcohol acyltransferase.

The terpenoid secondary metabolism generally has the characteristics of substrate and enzyme diversity. One enzyme can catalyze the same or multiple substrates, and different synthases can also act on the same substrate to produce multiple products [23]. The biosynthesis of terpenoids is mainly divided into the following three stages: (i) the generation stage of the C5 precursor isopentyl diphosphate (IPP) and its double-bond isomer dimethylallyl diphosphate (DMAPP); (ii) the generation stage of direct precursors such as geranyl diphosphate (GPP), farnesyl diphosphate (FPP), and geranyl geranyl diphosphate (GGPP); (iii) the terpene generation and modification stage. The current research is relatively clear on the first two stages. Terpenoids are produced in plants by two independent pathways, namely, the mevalonate (MVA) pathway and methylerythritol phosphate (MEP) pathway, both of which involve the C5 precursor IPP and its allyl isomer DMAPP [9]. The mevalonate pathway and the methylerythritol pathway are involved in this pathway but are not completely independent (Figure 2a). The main method of terpenoid synthesis is to synthesize IPP and the C5 precursor. Then, as the general framework for terpenoid syntheses, a series of precursor substances is used for catalysis. Finally, the modification stage produces the corresponding derivative.

In the MVA pathway, the acetyl CoA is first formed as acetoacetyl-CoA under the catalysis of acetyl CoA acetyl transferase (AACT) catalysis. Then, under the catalysis of hydroxymethylglutaryl CoA synthase (HMGCS), the acetoacetyl-CoA is converted to the 3-hydroxy3-methylglutaryl coenzyme A (HMG-CoA). Subsequently, HMG-CoA is catalyzed by two molecules of NADPH and hydroxymethylglutaryl-CoA reductase (HMGCR) to generate MVA, which is then converted to mevalonate-5-phosphate (MVAP) under the catalysis of mevalonate kinase (MVK). Then, under the catalysis of phosphomevalonate kinase (PMK), MVAP is converted to mevalonate-5-diphosphate (MVAPP), and, finally, MVAPP is generated under the catalysis of mevalonate diphosphomevalonate decarboxylase (MVD).

In the MEP pathway, pyruvate and glyceraldehyde-3-phosphate first generate 1-deoxy-D-xylose 5-phosphate (DXP) under the action of 1-deoxy-D-xylose 5-phosphate synthase (DXS), and then DXP forms 2C-Methyl-D-erythritol 4-phosphate (MEP) under the catalysis of 1-deoxy-Dlxylose 5-phosphate reductoisomerase (DXR). Through the 2C methyl-D erythritol 4-phosphate cytidine transferase (MCT) catalysis, MEP is converted to 2C-methyl-D-erythrin-4-cytidine diphosphate, followed by 4-diphosphocytidyl-2-C-methyl-D-erythritol kinase (CMK) catalysis to produce 2-C-methyl-D-erythrin-4-cytidine diphosphate. Then, under the action of 2-C-methyl-D-erythritol 2,4-cyclodiphosphate synthase (MDS), 2C-methyl-D-erythritol 2,4-cyclic diphosphate is generated, which is catalyzed by 4-hydroxy-3-methylbutene-2-enyl-phosphate synthase (HDS) to form 1-hydroxy-2-methyl-2-(E)-butenyl 4-diphosphate (HMBPP). Finally, HMBPP forms isopentenyl diphosphate (IPP) under the catalysis of 4-hydroxy-3-methylbuten-2-en-1-yl diphosphate reductase (HDR). It is interesting that IPP is partially converted into the double-bond isomer 1-hydroxy-2-methyl-2-(E)-butenyl 4-diphosphate (DMAPP) under the action of isopentyl diphosphate isomerase (IDI), which are the C5 precursor substances of all terpenoid compounds. In the fruit, IPP is mainly synthesized through the MEP pathway.

The C5 precursor IPP and DMAPP further generate the direct precursors of terpenoids under the catalysis of isopentenyltransferase: GPP, FPP, and GGPP. The direct precursor geranyl diphosphate (GPP) of monoterpenes is synthesized from one molecule of IPP and one molecule of DMAPP under the catalysis of geranyl diphosphate synthase (GPPS). The direct precursor of sesquiterpenes, FPP, is generated through a two-step condensation reaction of two molecules of IPP and one molecule of DMAPP catalyzed by farnesyl diphosphate synthase (FPPS). The direct precursor of diterpenes, GGPP, is generated through a three-step condensation reaction of three molecules of IPP and one molecule of DMAPP catalyzed by geranylgeranyl diphosphate synthase (GGPPS). Methyltransferase is one of the important enzymes in this pathway, helping to catalyze and generate more aroma substances or further enhancing the expression of aroma compounds based on the original aroma of aromatic plants. Some studies found that the key enzyme involved in volatile terpenoid synthesis is terpenoid synthetase (TPS), and TPS is an important terminal enzyme that catalyzes the formation of linalsol by GPP as a substrate [23]. Linalsol is a linear monoterpene alcohol widely present in more than 200 plants and is a key odor substance affecting fruit aroma [24,25].

The energy flow and distribution of MVA in different fruit trees or organs are different under the two pathways, and the two pathways produce different precursor substances (GPP or FPP), subsequently resulting in the diverse structures of terpenes [26,27]. Molecular breeding technology can enhance plants by regulating the content of terpenoids in transgenic plants. From the perspective of biosynthesis and transcriptional regulation, there is competition between the different branches of the metabolism for the same precursor [28]. For example, *GGPPS* genes are located at the intersection of the terpene metabolic pathways. They not only participate in the synthesis of monoterpenes and sesquiterpenes, but also promote the accumulation of aroma compounds in fruits and catalyze the formation of carotenoid precursors. By regulating the *GGPPS* gene, it may be possible to simultaneously regulate the production of products from both pathways and co-ordinate the metabolite flux between the monoterpene and carotenoid biosynthesis pathways, thereby affecting the formation of fruit color and aroma [29].

### 3.2. Phenylpropane Synthesis Pathway

The phenylpropane synthesis pathway is an anabolic pathway that requires complex steps and the biosynthesis of multiple genes (Figure 2b). This metabolic pathway is influenced not only by temporal and spatial regulation during the plant growth and development but also by the biosynthesis of precursor substances. Under the catalysis of enzymes, trans-cinnamic acid, through a series of methylation reactions, eventually produces a variety of benzene ring compounds and some benzene derivatives, which constitute the main components of plant flowers [30]. These compounds are volatile and contribute to the unique aroma of the plant. To date, research on the metabolic mechanism of related compounds produced by this pathway and the key enzymes involved is still in the initial stage. Only some progress has been made in understanding the mechanism of this modification.

### 3.3. Fatty Acid Synthesis Pathway

Fatty acid-derived aroma volatiles are metabolized mainly through the lipoxygenase (LOX) pathway and the α-/β-oxidation pathway, in which unsaturated fatty acids such as linoleic acid and linolenic acid are the core precursors [4]. In peach, the key enzymes such as LOX, hydroperoxidase lyase (HPL), ethanol dehydrogenase (ADH), and alcohol acyltransferase (AAT) have been shown to be involved in this metabolic process [31] (Figure 2c). Alcohols, as precursors to the synthesis of ester aroma compounds in plants, are catalyzed by AATs to convert various alcohols produced by fatty acid and amino acid pathways into ester aromas [32]. Lactones in plants are derived from fatty acids and are formed through dehydrogenation, epoxidation, hydration, hydroxylation, β-oxidation, and internal esterification with hydroxy-acetyl-CoA [33,34,35]. The key enzymes such as fatty acid desaturase (FAD), epoxide hydrolase (EPH), and acyl-CoA oxide (ACX) play crucial roles in this process [33,34].

Fatty acid derivatives include several small alcohols and aldehydes, and fatty acid oxidase is the main regulatory enzyme (Figure 2c). At the same time, volatile unsaturated fatty acids are also one of the main sources of aroma in vegetables, melons, and fruits. For example, the metabolism of major VOCs in pear aroma products is dependent on the fatty acid oxidase pathway [36]. However, there are differences in the fruit aroma components among grape varieties, and there are fatty acid substances such as ethyl acetate [37]. Some esters, alcohols, and acids were identified in the fruit aroma of passion fruit, and it was found that esters were the main components of the volatile aroma of passion fruit. Ethyl butyrate, ethyl caproate, and propyl acetate have shown high odor importance in fruits [38]. The expression of lipoxygenase in passion fruit has been shown to have a positive regulatory effect [39].

## 4. Regulation of Key Genes Involved in Aroma Formation

### 4.1. Structural Genes

During the whole development process of plants, the synthesis of volatile compounds differs in time and space, and these differences are often the result of changes in enzyme levels and activities [40]. This complexity is also reflected in the large number of genes encoding secondary metabolic enzymes, which account for approximately 15–25% of the 20,000–60,000 genes in the plant genome [41]. Through correlation and gene expression analysis, Sanchez et al. [33] identified candidate genes related to aroma synthesis in peach fruit and reported that the *PPN018G03* gene may affect the biosynthesis of phenylacetaldehyde through the transaminase pathway, thus affecting peach aroma. The *PPN023E05* and *PPN052H12* genes may regulate aroma synthesis by regulating the degradation pathway of fatty acids, and the fatty acid desaturase encoded by the *PPN052H12* gene catalyzes the generation of unsaturated fatty acids to avoid entering the LOX/HPL pathway to produce undesirable aromas. Using RNA-seq, qRT-PCR, and other technologies, Li et al. [42] reported that ppa002510 and ppa002282 may be two important genes related to gluconolactone biosynthesis in the peach *ACX* gene family. With the development and maturation of passion fruit, the expression level of PELOX4, LOX enzyme activity, and total ester content all increase [39]. Based on the metabolomics and transcriptomic analysis of strawberry fruits, 27 differentially expressed genes (DEGs) were found to be closely related to ester and lactone synthesis. These genes belong to the *AAT*, *LOX*, *ADH*, and cytochromatin P450 subfamilies, and may influence the production of aroma precursors [43]. In the biosynthesis of C-decalactone, AATs with esterification ability and ACX enzyme activity play a key role. Among AAT enzymes, PpAAT1 exhibits stronger esterification ability compared to the other AAT enzymes [35]. Changes in the expression of MdArAT, MdACPD, MdADH3, MdAAT2, and MdLOX may be the reason for the differences in the VOCs among different apple varieties [44].

### 4.2. Transcription Factors (TFs)

Transcriptional regulation plays an important role in the regulation of plant genome expression, especially in the production of fruit aroma [4]. TFs play an important role in regulating the synthesis of esters, alcohols, aldehydes, eugenol, terpenes, and benzene/phenylc aromas [45]. Wei et al. [46] investigated the synthesis and regulatory mechanism of volatile linalool during peach fruit maturation. They concluded that the transcription factor PpERF61 can bind to the promoters of the *PpTPS1* and *PpTPS3* genes to activate their expression. In addition, another promoter, PpbHLH1, can interact with PpERF61 to activate *PpTPS3* gene expression. In mature strawberry fruits, FaDOF2 is a key transcription factor that regulates the production of the phenylpropanoid volatile compound eugenol. It also co-regulates the expression of the *FaEGS2* gene with the *FaEOBII* gene, thereby promoting the production of eugenol. In addition, the transcription factor FaMYB10 can further affect eugenol content by activating the *FaEOBII* gene [47]. In peach, linalsol, an important aroma-contributing compound, is synthesized by the terpenoid synthases *PpTPS1* and *PpTPS3*, and the TF *PpbHLH1* can directly bind to the promoter of the *PpTPS3* gene to activate its expression. The AP2/ERF family TF *PpERF61* can bind the DRE/CRT motif in the *PpTPS1* and *PpTPS3* promoters to activate their expression, thereby promoting the production of linalool [25,46].

Wang et al. [43] predicted the diploid strawberry *Fragaria nilgerrensis* Schlecht in the wild. There are 35 TFs associated with the aroma formation, including members from the *bHLH*, *MYB*, *bZIP*, *NAC*, *AP2*, *GATA*, and *TCP* family. Sheng et al. [48] cultivated strawberry *Fragaria × ananassa* Duch. A total of 498 members of the AP2/ERF superfamily were predicted in the genome, and the *FaAP2* gene was found to be a key TF regulating aroma production. In the ripening process of apples, peaches, and tomatoes, *NAC* TFs can directly target ester synthesis genes and promote the accumulation of ester substances [49]. In addition, the TFs *PuWRKY24*, *PuIAA29*, and *PuTINY* are associated with aroma formation in ‘Nanguo’ pear (*Pyrus ussuriensis* Maxim.) during the fruit ripening [36]. Liu et al. [50] studied the impact of jasmonate application on the synthesis of volatile compounds in apples. They have found that, after the jasmonate treatment, the expression of the transcription factor *MdMYC2* in apples increased, leading to a significant enhancement in the volatile compound synthesis.

### 4.3. Noncoding RNAs

In addition to the widely known coding RNAs involved in transcriptional regulation, small RNA (smRNA) sequences, such as microRNAs (miRNAs) and long noncoding RNAs (lncRNAs), which do not have protein coding potential, are also key regulators of gene expression, RNA processing, and translation at the transcriptional level [51,52]. miRNAs are endogenous ncRNAs that regulate gene expression at the post-transcriptional level by binding to target mRNAs [53,54]. miRNAs regulate fruit softening and aroma biosynthesis during banana ripening [55]. Shi et al. [56] identified differentially expressed miRNAs, including mdm-miR172a-h, mdm-miR159a/b/c, and mdm-miR160a-e, in response to low temperature during the formation of the ‘Nanguo’ pear aroma. These miRNAs bind to their respective target genes, playing an important regulatory role in the process of flavor weakening in the ‘Nanguo’ pear stored in cold conditions. lncRNAs represent a class of RNA transcripts longer than 200 nucleotides (nt) with no identifiable protein-coding potential. lncRNAs participate in the fruit development and ripening process of tomatoes, strawberries, and peaches. Compared with those in coding RNAs, lncRNAs in peach fruits exhibit lower expression levels, lower complexity of variable splicing, shorter isomers, and fewer exons [57]. Expression analysis has shown that lncRNAs in peach fruit have lower expression levels and fewer exons [57]. The 575 differentially expressed lncRNAs were divided into six clusters, which may be related to physiological and metabolic changes associated with the fruit ripening, such as the flavonoid biosynthesis, fruit texture softening, chlorophyll decomposition, and the accumulation of aroma compounds [57].

## 5. Epigenetic Factors

VOC release is highly dependent on the environment [58] and is also influenced by the biological clock, as well as epigenetic factors such as DNA methylation and histone modification. These factors can regulate the release of VOCs by controlling genes and TFs. In a sense, the biological clock is also epigenetic. This changes the daily VOC emissions of fruit [59]. Epigenetic regulation produces changes in gene function that can be inherited during mitosis or meiosis without the need for DNA sequence polymorphisms [60]. Methylation and histone modification have been shown to epigenetically regulate the various nonvolatile phenylpropane-like biosynthesis pathway genes [59].

Related studies [2,4,49] have shown that promoters of the *NAC* and *AAT* genes related to the synthesis of volatile compounds are widely bound by the inhibitory methylation marker H3K27me3 during the ripening process of peach and apple. The levels of *H3K27me3* decrease with fruit ripening, thereby activating the expression of the *NAC* and *AAT* genes. The synthesis of volatile ester compounds can be promoted [49]. During peach fruit ripening, the DNA methylation level of the *PpTPS3* gene promoter decreases, which is associated with increased *PpTPS3* gene expression and increased linalool content [25]. In a subsequent study, Wei et al. [46] reported that, during the ripening process of peach fruit, the expression of the transcription factor PpERF61 was negatively correlated with the DNA methylation level of its promoter region (R = −0.892, *p* < 0.01). That is, a decrease in the DNA methylation level is also correlated with an increase in PpERF61 expression and the linalool content [46]. In addition, RNA-based epigenetic mechanisms can also modify chromatin and silence transcription, but these mechanisms are poorly understood in fruit trees.

## 6. Main Factors Affecting Fruit Aroma Synthesis

Fruit aroma is influenced by a variety of factors, including intrinsic genetic factors [61], plant hormones [62], the cultivation environment [63], cultivation practices [64], and postharvest storage [65]. There are differences in the aroma substances in fruits of the same citrus variety from different regions. Cheong et al. [66] reported that the total amount of aroma substances in the fruit juice of Kaman oranges from Vietnam was three times greater than that in the juice of oranges from Malaysia and the Philippines. The types and contents of strawberry aroma substances under soil cultivation were significantly greater than those under substrate cultivation [67]. The application of selenium fertilizer has a significant positive effect on tomato aroma [68]. The ‘Salustiana’ and ‘Navelina’ orange juices from organic and conventional cultivation systems are rich in different types of aroma substances [64]. Therefore, studying the influence of cultivation conditions on the metabolism of citrus fruit aroma substances can improve the aroma quality of citrus fruits.

Hydrogen nanobubble water (HNW) treatment before harvest can alleviate the negative effects of fertilizer on strawberry fruit aroma. The content of flavor compounds, such as esters (e.g., ethyl acetate), acids (e.g., acetic acid), and soluble sugars (glucose, fructose, sucrose, etc.), can also be increased with treatment [69]. Tietel et al. [70] reported that, during storage, the contents of 31 aroma substances in ‘Mor’ broad-peel citrus fruits decreased more than 50%, while the contents of 13 aroma substances increased more than twofold. The loss of the former aroma substances was due to the degradation of monoterpenes and sesquiterpenes. The latter increase may be due to the metabolites of alcohol fermentation, fatty acid derivation, and amino acid breakdown. In addition, the contents of aroma substances in the fruits of ‘Navelina’ sweet orange, ‘Clemenules’ ponkan, ‘Prenules’ ponkan, and ‘Basol’ ponkan are not affected by ethylene treatment. However, after ethylene treatment of ‘Oronules’ and ‘Clemenrubi’ fruit, the content of esters, such as ethyl propionate and ethyl caprylate, in the fruits of Ponkan increased [65]. Storage temperature and ethylene content are both factors that affect the aroma of fruits after harvest. Low temperature and controlled ethylene concentration can delay the ripening of fruits and subsequently affect the aroma of horticultural crops. Compared with treatment at the beginning of picking, the application of the ethylene and 1-methylcyclopropene (1-MCP) not only preserved the freshness of stored blueberry fruits but also did not have a significant impact on aroma substances [71]. The degradation effect of ethylene was effectively neutralized by 1-MCP. Compared with the CK, 1-MCP with or without ethylene increased tightness, functional component content, antioxidant activity, and inhibited the ethylene production and respiration rate. Therefore, the simultaneous use of ethylene and 1-MCP treatment guaranteed better quality and volatile compounds in blueberry fruit during the whole storage [71]. Luo et al. [62] studied the effect of methyl jasmonate on the aroma quality of refrigerated ‘Nanguo’ pear. The results showed that the application of methyl jasmonate can affect LOX pathway-related genes and TFs, helping to prevent refrigeration-induced ester synthesis and improve pear quality. Further studies have shown that methyl jasmonate treatment activated the biosynthetic pathways of LOX, methylerythritol phosphate (MEP), and shikimate in pear skin and pulp.

## 7. Research on Flavor Quality Improvement

Quantitative trait loci (QTLs) play an important role in the biosynthesis of fruit aroma substances, and those related to aroma quality can be accurately located by constructing molecular linkage maps. This approach can significantly enhance the characterization and assessment of the genetic capacity for improving the aroma quality [72,73]. Fan et al. [74] have used octoploid cultivated strawberry to demonstrate how genomic heterozygosity, transcriptomic intricacy, and fruit metabolomic diversity can be treated as strengths and leveraged to uncover fruit flavor genes and their regulatory elements. Yang et al. [72] used the QTL method to further explore the genetic basis of apple aroma traits, and successfully identified 87 QTLs associated with 15 volatile compounds in 14 linkage groups. Additionally, they found a specific group of QTLs in the sixth linkage group that was closely related to the yield of ester substances. Further studies have shown that the MdAAT6 gene within the QTL localization interval plays a key role in the biosynthesis of ester substances [72]. Combined with aroma data and the constructed genetic linkage map, a candidate gene *AAT6* was detected in the QTL location region. This gene led to a significant increase in the accumulation of esters in fruits after overexpression. Multi-omics datasets and comprehensive genome-wide association studies involving over 300 individuals in strawberry provide effective reference for the subsequent expression of aroma-related specific genes. Therefore, the identification of QTLs for strawberry and apple provides new insights for the genetic improvement of aroma traits.

The flavor of fresh fruit is influenced by both the outside and the inside of the fruit. Fruit appearance, such as its size, shape, color, and nutritional composition, gives the fruit a unique visual appearance. The internal sweetness, acidity, hardness, and volatile aromatic components determine the taste and depth of flavor [5,7]. Studies have shown that the content and diversity of lactone compounds in soft peaches are greater than those in firm peaches. In contrast, crisp and firm peaches may not have some of the important aroma qualities. Therefore, determining the correlation between aroma and other fruit traits is essential for promoting selective breeding and obtaining high-quality fruit varieties. By understanding the correlation between the aroma and characteristic traits of each fruit, targeted breeding work is expected to develop high-quality fruit varieties that excel in terms of aroma and other desirable traits in the future.

## 8. Issues and Prospects

Fruit aroma is an important indicator of fruit quality. With the improvement of consumer living standards, the demand for high-quality fruit has increased. To date, although transcriptomics and metabolomics technologies provide important gene and differentially abundant metabolite data of specific aroma components for the study of fruit aroma, they still face some challenges in practical application, and most fruits lack stable genetic transformation systems. The fruit-bearing period of some fruit trees is long, and the aroma of fruit is challenging to quantify through taste expression. It is easily affected by environmental factors and agronomic measures. Furthermore, how the key node genes regulating aroma synthesis in the synthesis pathway co-ordinate the substrate concentration levels of the different metabolic branches to directly or indirectly participate in the formation of aroma compounds needs to be further explored. Analysis at the protein level has application value in the improvement of fruit aroma traits. Developing methods to find the active sites of residues near the substrate-binding zone based on the enzyme engineering of the protein structure, and the construction of a more reliable mutation library to obtain better catalytic efficiency and specificity, facilitate the formation of aroma components, and improve the aroma content of fruits, is crucial.

The main challenge for the future is to determine not only which VOCs contribute to fruit flavor but also, and more importantly, which VOCs contribute to consumer preferences. The solution to this problem is molecular breeding, which reduces complex traits into a set of molecular markers that can be explored in depth through GWASs. In future studies, it is crucial to comprehensively apply and analyze the significant findings of genomics, proteomics, transcriptomics, and metabolomics related to aroma formation. A multi-omics approach should be used to further analyze the multilevel molecular regulatory network of aroma formation. The function of key genes should be verified through the instantaneous or stable genetic transformation to reveal their specific mechanisms of action. In this study, the main aroma components and synthetic pathways of fruits were summarized. The important molecular regulatory mechanisms of aroma synthesis and important research on flavor improvement were reviewed to provide valuable insights for enhancing fruit aroma quality and molecular breeding.

## 9. Conclusions

Aroma is produced by the mixing of various volatile aromatic compounds and is an important factor affecting the flavor and quality of fruits, which largely determines the purchasing desire of consumers. There are significant differences in the causes, biosynthesis pathways, and content of different fruit aroma substances. This article summarizes the pathways and potential regulatory mechanisms of the synthesis of major volatile components in fruits, outlines the main reasons affecting fruit aroma formation and related research on improving the flavor quality, and finally looks forward to possible future research directions. This review provides theoretical references for improving the flavor and quality of fruits.

## Figures and Tables

**Table 1:** 10 Aroma components of representative fruits.

Species	Main Aroma Component	Variety of Aroma Substance	Reference
Apple	N-butyl 2-methylacetate, hexyl acetate	300	Qin et al. [16]
Kiwifruit	ethyl butyrate, (E)-2-hexene-1-ol, (E)-2-hexenal	172	Liang et al. [18]
Grape	ethyl butyrate, ethyl hexanoate, octanal, nonanal, limonene	26	Zhu et al. [19]
Peach	hexanal, (Z)-3-hexene-1-ol, (E)-2-hexenal, 3-mercaptohexanol, nonanal	100	Li et al. [20]
Banana	2-methylpropyl butyrate, orthonitrophenyl-beta-d-fucopyranoside	48	Pino and Febles [17]
Tomato	cis-3-hexenal, trans-2-hexenal, cis-3-hexenol	400	Buttery and Takeoka [21]
Mango	terpene olefins, 3-pinene, caryophyllene, α-pinene	60	Yi et al. [22]
Pear	hexyl acetate, alpha-farnesene	202	Wang et al. [6]
Citrus	limonene, citral, naringene	162	Zhang et al. [8]
Strawberry	methyl butyrate, ethyl butyrate, butyl acetate, methyl caproate, ethyl hexanoate	979	Ulrich et al. [7]

## Data Availability

The original contributions presented in the study are included in the article, further inquiries can be directed to the corresponding authors.

## References

[1] Hou X., Jiang J., Luo C., Rehman L., Li X., Xie X. (2023). Advances in detecting fruit aroma compounds by combining chromatography and spectrometry. J. Sci. Food Agric..

[2] El Hadi M.A., Zhang F.J., Wu F.F., Zhou C.H., Tao J. (2013). Advances in fruit aroma volatile research. Molecules.

[3] Wang Q.H., Wang L., Wu W.J., Guo J.X., Shen Y.Y., Wu G.L. (2021). Advances in aroma compounds biosynthesis and hormone regulation of fruit. Mol. Plant Breed..

[4] Li X., Gao P., Zhang C., Xiao X., Chen C., Song F. (2023). Aroma of peach fruit: A review on aroma volatile compounds and underlying regulatory mechanisms. Int. J. Food Sci. Technol..

[5] Chen X., Quek S.Y. (2023). Free and glycosidically bound aroma compounds in fruit: Biosynthesis, transformation, and practical control. Crit. Rev. Food Sci. Nutr..

[6] Wang X., Chen Y., Zhang J., Wang Z., Qi K., Li H., Tian R., Wu X., Qiao X., Zhang S. (2023). Comparative analysis of volatile aromatic compounds from a wide range of pear (*Pyrus* L.) germplasm resources based on HS-SPME with GC-MS. Food Chem..

[7] Ulrich D., Kecke S., Olbricht K. (2018). What do we know about the chemistry of strawberry aroma?. J. Agric. Food Chem..

[8] Zhang H., Xie Y., Liu C., Chen S., Hu S., Xie Z., Deng X., Xu J. (2017). Comprehensive comparative analysis of volatile compounds in citrus fruits of different species. Food Chem..

[9] Dudareva N., Klempien A., Muhlemann J.K., Kaplan I. (2013). Biosynthesis, function and metabolic engineering of plant volatile organic compounds. New Phytol..

[10] Lewinsohn E., Sitrit Y., Bar E., Azulay Y., Ibdah M., Meir A., Yosef E., Zamir D., Tadmor Y. (2005). Not just colors—Carotenoid degradation as a link between pigmentation and aroma in tomato and watermelon fruit. Trends Food Sci. Technol..

[11] Siboza X.I., Bertling I., Odindo A.O. (2014). Salicylic acid and methyl jasmonate improve chilling tolerance in cold-stored lemon fruit (*Citrus limon*). J. Plant Physiol..

[12] Guo Y.F., Zhang Y.L., Shan W., Cai Y.J., Liang S.M., Chen J.Y., Lu W.J., Kuang J.F. (2018). Identification of two transcriptional activators MabZIP4/5 in controlling aroma biosynthetic genes during banana ripening. J. Agric. Food Chem..

[13] Forney C.F., Qiu S., Jordan M.A., McCarthy D., Fillmore S. (2022). Comparison of volatile compounds contributing to flavor of wild lowbush (*Vaccinium augustifolium*) and cultivated highbush (*Vaccinium corymbosum*) blueberry fruit using gas chromatography-olfactometry. Foods.

[14] Muhlemann J.K., Klempien A., Dudareva N. (2014). Floral volatiles: From biosynthesis to function. Plant Cell Environ..

[15] McGarvey D.J., Croteau R. (1995). Terpenoid metabolism. Plant Cell.

[16] Qin L., Wei Q.P., Kang W.H., Zhang Q., Sun J., Liu S.Z. (2017). Comparison of volatile compounds in ‘Fuji’apples in the different regions in China. Food Sci. Technol. Res..

[17] Pino J.A., Febles Y. (2013). Odour-active compounds in banana fruit cv. Giant Cavendish. Food Chem..

[18] Liang Z., Fang Z., Pai A., Luo J., Gan R., Gao Y., Lu J., Zhang P. (2022). Glycosidically bound aroma precursors in fruits: A comprehensive review. Crit. Rev. Food Sci. Nutr..

[19] Zhu J., Xiao Z. (2019). Characterization of the key aroma compounds in peach by gas chromatography–olfactometry, quantitative measurements and sensory analysis. Eur. Food Res. Technol..

[20] Li L., Ma X.W., Zhan R.L., Wu H.X., Yao Q.S., Xu W.T., Luo C., Zhou Y.G., Liang Q.Z., Wang S.B. (2017). Profiling of volatile fragrant components in a mini-core collection of mango germplasms from seven countries. PLoS ONE.

[21] Buttery R.G., Takeoka G.R. (2004). Some unusual minor volatile components of tomato. J. Agric. Food Chem..

[22] Yi X.K., Liu G.F., Rana M.M., Zhu L.W., Jiang S.L., Huang Y.F., Lu W.M., Wei S. (2016). Volatile profiling of two pear genotypes with different potential for white pear aroma improvement. Sci. Hortic..

[23] Vranová E., Coman D., Gruissem W. (2012). Structure and dynamics of the isoprenoid pathway network. Mol. Plant.

[24] Tian H., Wang P., Zhan P., Yan H., Zhou W., Zhang F. (2017). Effects of β-glucosidase on the aroma characteristics of flat peach juice as assessed by descriptive sensory analysis and gas chromatography and compared by partial least squares regression. LWT Food Sci. Technol..

[25] Wei C., Liu H., Cao X., Zhang M., Li X., Chen K., Zhang B. (2021). Synthesis of flavour-related linalool is regulated by PpbHLH1 and associated with changes in DNA methylation during peach fruit ripening. Plant Biotechnol. J..

[26] Henry L.K., Gutensohn M., Thomas S.T., Noel J.P., Dudareva N. (2015). Orthologs of the archaeal isopentenyl phosphate kinase regulate terpenoid production in plants. Proc. Natl. Acad. Sci. USA.

[27] Tholl D. (2015). Biosynthesis and biological functions of terpenoids in plants. Adv. Biochem. Eng. Biotechnol..

[28] Sun Q., He L., Sun L., Xu H.Y., Fu Y.Q., Sun Z.Y., Zhu B.Q., Duan C.Q., Pan Q.H. (2023). Identification of SNP loci and candidate genes genetically controlling norisoprenoids in grape berry based on genome-wide association study. Front. Plant Sci..

[29] Barja M.V., Ezquerro M., Beretta S., Diretto G., Florez-Sarasa I., Feixes E., Fiore A., Karlova R., Fernie A.R., Beekwilder J. (2021). Several geranylgeranyl diphosphate synthase isoforms supply metabolic substrates for carotenoid biosynthesis in tomato. New Phytol..

[30] Peled-Zehavi H., Oliva M., Xie Q., Tzin V., Oren-Shamir M., Aharoni A., Galili G. (2015). Metabolic engineering of the phenylpropanoid and its primary, precursor pathway to enhance the flavor of fruits and the aroma of flowers. Bioengineering.

[31] Muto A., Müller C.T., Bruno L., McGregor L., Ferrante A., Chiappetta A.A.C., Bitonti M.B., Rogers H.J., Spadafora N.D. (2020). Fruit volatilome profiling through GC × GC-ToF-MS and gene expression analyses reveal differences amongst peach cultivars in their response to cold storage. Sci. Rep..

[32] Schwab W., Davidovich-Rikanati R., Lewinsohn E. (2008). Biosynthesis of plant-derived flavor compounds. Plant J..

[33] Sanchez G., Venegas-Calerón M., Salas J.J., Monforte A., Badenes M.L., Granell A. (2013). An integrative “omics” approach identifies new candidate genes to impact aroma volatiles in peach fruit. BMC Genom..

[34] Zhang L., Li H., Gao L., Qi Y., Fu W., Li X., Zhou X., Gao Q., Gao Z., Jia H. (2017). Acyl-CoA oxidase 1 is involved in γ-decalactone release from peach (*Prunus persica*) fruit. Plant Cell Rep..

[35] Peng B., Yu M., Zhang B., Xu J., Ma R. (2020). Differences in PpAAT1 activity in high- and low-aroma peach varieties affect γ-decalactone production. Plant Physiol..

[36] Li X., Qi L., Zang N., Zhao L., Sun Y., Huang X., Wang H., Yin Z., Wang A. (2022). Integrated metabolome and transcriptome analysis of the regulatory network of volatile ester formation during fruit ripening in pear. Plant Physiol. Biochem..

[37] Flamini R. (2005). Some advances in the knowledge of grape, wine and distillates chemistry as achieved by mass spectrometry. J. Mass Spectrom..

[38] Janzantti N.S., Monteiro M. (2017). HS-GC-MS-O analysis and sensory acceptance of passion fruit during maturation. J. Food Sci. Technol..

[39] Huang D., Ma F., Wu B., Lv W., Xu Y., Xing W., Chen D., Xu B., Song S. (2022). Genome-wide association and expression analysis of the lipoxygenase gene family in *Passiflora edulis* revealing *PeLOX4* might be involved in fruit ripeness and ester formation. Int. J. Mol. Sci..

[40] Gibney E.R., Nolan C.M. (2010). Epigenetics and gene expression. Heredity.

[41] Pichersky E., Gang D.R. (2000). Genetics and biochemistry of secondary metabolites in plants: An evolutionary perspective. Trends Plant Sci..

[42] Li X.W., Jiang J., Zhang L.P., Yu Y., Ye Z.W., Wang X.M., Zhou J.Y., Chai M.L., Zhang H.Q., Arús P. (2015). Identification of volatile and softening-related genes using digital gene expression profiles in melting peach. Tree Genet. Genomes.

[43] Wang A.H., Ma H.Y., Zhang B.H., Mo C.Y., Li E.H., Li F. (2022). Transcriptomic and metabolomic analyses provide insights into the formation of the peach-like aroma of *Fragaria nilgerrensis* Schlecht. Fruits. Genes.

[44] Feng S., Yan C., Zhang T., Ji M., Tao R., Gao H. (2021). Comparative study of volatile compounds and expression of related genes in fruit from two apple cultivars during different developmental stages. Molecules.

[45] Li L.X., Fang Y., Li D., Zhu Z.H., Zhang Y., Tang Z.Y., Li T., Chen X.S., Feng S.Q. (2023). Transcription factors MdMYC2 and MdMYB85 interact with ester aroma synthesis gene *MdAAT1* in apple. Plant Physiol..

[46] Wei C., Li M., Cao X., Jin Z., Zhang C., Xu M., Chen K., Zhang B. (2022). Linalool synthesis related PpTPS1 and PpTPS3 are activated by transcription factor PpERF61 whose expression is associated with DNA methylation during peach fruit ripening. Plant Sci..

[47] Molina-Hidalgo F.J., Medina-Puche L., Cañete-Gómez C., Franco-Zorrilla J.M., López-Vidriero I., Solano R., Caballero J.L., Rodríguez-Franco A., Blanco-Portales R., Muñoz-Blanco J. (2017). The fruit-specific transcription factor FaDOF2 regulates the production of eugenol in ripe fruit receptacles. J. Exp. Bot..

[48] Sheng L., Ni Y., Wang J., Chen Y., Gao H. (2021). Characteristic-aroma-component-based evaluation and classification of strawberry varieties by aroma type. Molecules.

[49] Cao X., Wei C., Duan W., Gao Y., Kuang J., Liu M., Chen K., Klee H., Zhang B. (2021). Transcriptional and epigenetic analysis reveals that NAC transcription factors regulate fruit flavor ester biosynthesis. Plant J..

[50] Liu X., Feng Y., Li S., Li D., Yu J., Zhao Z. (2023). Jasmonate-induced MdMYC2 improves fruit aroma during storage of ‘Ruixue’ apple based on transcriptomic, metabolic and functional analyses. LWT Food Sci. Technol..

[51] Moazed D. (2009). Small RNAs in transcriptional gene silencing and genome defence. Nature.

[52] Cech T.R., Steitz J.A. (2014). The noncoding RNA revolution-trashing old rules to forge new ones. Cell.

[53] Mallory A.C., Vaucheret H. (2006). Functions of microRNAs and related small RNAs in plants. Nat. Genet..

[54] Meyers B.C., Axtell M.J., Bartel B., Bartel D.P., Baulcombe D., Bowman J.L., Cao X., Carrington J.C., Chen X., Green P.J. (2008). Criteria for annotation of plant MicroRNAs. Plant Cell.

[55] Lakhwani D., Sanchita, Pandey A., Sharma D., Asif M.H., Trivedi P.K. (2020). Novel microRNAs regulating ripening-associated processes in banana fruit. Plant Growth Regul..

[56] Shi F., Zhou X., Yao M.M., Tan Z., Zhou Q., Zhang L., Ji S.J. (2019). miRNAs play important roles in aroma weakening during the shelf life of ‘Nanguo’ pear after cold storage. Food Res. Int..

[57] Zhou H., Ren F., Wang X., Qiu K., Sheng Y., Xie Q., Shi P., Zhang J., Pan H. (2022). Genome-wide identification and characterization of long noncoding RNAs during peach (*Prunus persica*) fruit development and ripening. Sci. Rep..

[58] Raguso R.A. (2008). Wake up and smell the roses: The ecology and evolution of floral scent. Annu. Rev. Ecol. Evol. Syst..

[59] Picazo-Aragonés J., Terrab A., Balao F. (2020). Plant volatile organic compounds evolution: Transcriptional regulation, epigenetics and polyploidy. Int. J. Mol. Sci..

[60] DuPont C., Armant D.R., Brenner C.A. (2009). Epigenetics: Definition, mechanisms and clinical perspective. Semin. Reprod. Med..

[61] Ren J.N., Tai Y.N., Dong M., Shao J.H., Yang S.Z., Pan S.Y., Fan G. (2015). Characterisation of free and bound volatile compounds from six different varieties of citrus fruits. Food Chem..

[62] Luo M., Zhou X., Hao Y., Sun H., Zhou Q., Sun Y., Ji S.J. (2021). Methyl jasmonate pretreatment improves aroma quality of cold-stored ‘Nanguo’ pears by promoting ester biosynthesis. Food Chem..

[63] Teixeira A., Eiras-Dias J., Castellarin S.D., Gerós H. (2013). Berry phenolics of grapevine under challenging environments. Int. J. Mol. Sci..

[64] Cuevas F.J., Moreno-Rojas J.M., Ruiz-Moreno M.J. (2017). Assessing a traceability technique in fresh oranges (*Citrus sinensis* L. Osbeck) with an HS-SPME-GC-MS method. Towards a volatile characterisation of organic oranges. Food Chem..

[65] Sdiri S., Rambla J.L., Besada C., Granell A., Salvador A. (2017). Changes in the volatile profile of citrus fruit submitted to postharvest degreening treatment. Postharvest Biol. Technol..

[66] Cheong M.W., Zhu D., Sng J., Liu S.Q., Zhou W., Curran P., Yu B. (2012). Characterisation of calamansi (*Citrus microcarpa*). Part II: Volatiles, physicochemical properties and non-volatiles in the juice. Food Chem..

[67] Li Y., Zhang Y., Liu X., Xiao Y., Zhang Z., Shi Y., Kong W., Yang X., Jiang G., Zhang B. (2021). Cultivation conditions change aroma volatiles of strawberry fruit. Horticulturae.

[68] Meucci A., Shiriaev A., Rosellini I., Malorgio F., Pezzarossa B. (2021). Se-enrichment pattern, composition, and aroma profile of ripe tomatoes after sodium selenate foliar spraying performed at different plant developmental stages. Plants.

[69] Li L., Wang J., Jiang K., Kuang Y., Zeng Y., Cheng X., Liu Y., Wang S., Shen W. (2022). Preharvest application of hydrogen nanobubble water enhances strawberry flavor and consumer preferences. Food Chem..

[70] Tietel Z., Bar E., Lewinsohn E., Feldmesser E., Fallik E., Porat R. (2010). Effects of wax coatings and postharvest storage on sensory quality and aroma volatile composition of ‘Mor’ mandarins. J. Sci. Food Agric..

[71] Xu F., Liu Y., Dong S., Wang S. (2020). Effect of 1-methylcyclopropene (1-MCP) on ripening and volatile compounds of blueberry fruit. J. Food Process. Preserv..

[72] Yang S., Yu J., Yang H., Zhao Z. (2023). Genetic analysis and QTL mapping of aroma volatile compounds in the apple progeny ‘Fuji’ × ‘Cripps Pink’. Front. Plant Sci..

[73] Klee H.J., Tieman D.M. (2018). The genetics of fruit flavour preferences. Nat. Rev. Genet..

[74] Fan Z., Tieman D.M., Knapp S.J., Zerbe P., Famula R., Barbey C.R., Folta K.M., Amadeu R.R., Lee M., Oh Y. (2022). A multi-omics framework reveals strawberry flavor genes and their regulatory elements. New Phytol..

